# Augmenting anti-inflammatory macrophage function in colitis: a neuroimmune mechanism to drive intestinal wound repair

**DOI:** 10.1152/ajpgi.00368.2024

**Published:** 2024-12-23

**Authors:** Natalie Bhesania, Michael A. Schumacher

**Affiliations:** 1 Department of Pediatrics, University of Southern California Keck School of Medicine, Los Angeles, California, United States; 2 Division of Gastroenterology, Hepatology, and Nutrition, The Saban Research Institute, Children’s Hospital Los Angeles, Los Angeles, California, United States

Macrophages are one of the most abundant leukocytes found throughout the gut and play essential roles in gastrointestinal homeostasis (1, 2), tissue injury and infection (3, 4), and disease (1, 5, 6). They are versatile immune cells involved in a variety of cellular functions ranging from pro-inflammatory (e.g., cytokine secretion and antigen presentation) to pro-repair (e.g., growth factor secretion and phagocytosis) depending on tissue context. These functions are largely driven by discrete signals in the tissue microenvironment that drive cell polarization to a spectrum of activated subtypes (1).

Inflammatory bowel disease (IBD) is a family of chronic inflammatory disorders of the gastrointestinal tract where persistent and aggressive immune cell responses are believed to be a key driver of disease severity. In particular, pro-inflammatory macrophage accumulation is associated with IBD pathogenesis (7), and many studies have demonstrated that attenuating pro-inflammatory macrophage function ameliorates experimental disease (1). The pro-inflammatory effect of macrophages has been well documented in murine colitis and human IBD (8). However, less is known about the tissue repair roles of macrophages in IBD and whether altered anti-inflammatory function contributes to disease. A body of recent work has found that skewing macrophages to anti-inflammatory states may protect against intestinal inflammation. For instance, human macrophages polarized with interleukin-4 (IL-4) to an anti-inflammatory phenotype have been shown to promote wound repair in human colon-derived epithelial cells and to limit colonic inflammation in experimental colitis (9).

Although an anticolitic effect of IL-4-treated macrophages has been demonstrated, the precise signals in the complex tissue inflammatory environment of IBD that augment and fine-tune the function of these cells have remained largely unexplored. A recent paper published in the *American Journal of Physiology—Gastrointestinal and Liver Physiology* made a crucial advance in our understanding of how complex tissue signals can direct and amplify pro-reparative functions of macrophages in disease (10). Through an unbiased screen of isolated human macrophages that were polarized with IL-4, the investigators of this study identified novel cell surface receptors that are induced by treatment. Intriguingly, they discovered that the receptor for the neuronal signaling ligand, calcitonin gene-related peptide (CGRP), is highly elevated in these cells; an interesting finding consistent with a body of literature showing CGRP regulation of macrophages in multiple tissue types throughout the body (11, 12).

To investigate the role of CGRP in regulating human macrophage function, the authors performed an array of studies with CGRP and IL-4-treated macrophages focusing on known anti-inflammatory and pro-reparative functions of these cells. Notably, they found that CGRP stimulation of IL-4-treated macrophages substantially enhanced wound healing in epithelial cells, an effect likely driven by increased production of the growth factor transforming growth factor beta (TGFβ). Mechanistically, they identified cyclooxygenase-1 (COX-1) as the likely mediator of these effects. Furthermore, colitis studies showed that the transfer of CGRP + IL-4-treated macrophages significantly amplified the anticolitic effect observed versus the transfer of IL-4-treated macrophages alone. An important strength of this study in the relevance to potential therapeutics was the isolation of macrophages from both healthy subjects and Crohn’s disease patients. This strategy allowed authors to test if innate macrophage dysfunction in IBD could contribute to an impaired functional response. The finding that cells isolated from healthy and disease states equally show responsiveness to augmentation with CGRP underscores the potential suitability of targeting this pathway in patients with IBD.

The prevalence of IBD continues to rise and is estimated at between 2.4 and 3.1 million in the United States (13). The development of therapeutics for IBD is a rapidly evolving area of study and identifying targeted interventions that could enhance the current standard of care is greatly needed. The exact etiology of IBD remains elusive; however, therapeutics predominantly focus on broadly suppressing overall inflammation via cytokine inhibition. With the advent of modern biological treatments over the past two decades, there has been significant advancement in IBD treatment goals, with a move toward achieving endoscopic healing in addition to symptomatic control (14). The Selecting Therapeutic Targets in Inflammatory Bowel Disease initiative update (STRIDE-II) consensus helped establish a timeline for each measured therapeutic goal with the prevention of disability and long-term complications at the forefront (15). Although our arsenal of medication options for the treatment of patients with IBD has grown over the last decade, many patients do not achieve deep remission and lose response to treatment, thus necessitating a change in medical therapy. This highlights the need to improve our understanding of the underlying pathogenesis and identify new therapeutic targets to treat this complex and diverse patient population including the possibility of macrophage-focused interventions.

Our therapeutic arsenal of medications continues to evolve but given the complex pathophysiology of IBD, targeting multiple pathways is likely essential for the management of this lifelong disease for many patients. The therapeutic focus has shifted toward achieving deep mucosal healing to prevent future complications of the disease. This study highlights that targeting CGRP and CGRP-stimulated signaling pathways in macrophages may be a viable route toward achieving this goal by enhancing the pro-tissue repair functions of these cells to reduce intestinal inflammation. In summary, this work highlights the intricacies that exist between immune cells and neuronal signals in the complex IBD environment, advances our understanding of autologous macrophage transfer as a possible future therapy for patients with IBD, and suggests CGRP as a new focal point for potential development of targeted IBD treatments.

## References

[1] Hegarty LM, Jones GR, Bain CC. Macrophages in intestinal homeostasis and inflammatory bowel disease. Nat Rev Gastroenterol Hepatol 20: 538–553, 2023. doi:10.1038/s41575-023-00769-0. 37069320

[2] Sehgal A, Donaldson DS, Pridans C, Sauter KA, Hume DA, Mabbott NA. The role of CSF1R-dependent macrophages in control of the intestinal stem-cell niche. Nat Commun 9: 1272, 2018. doi:10.1038/s41467-018-03638-6. 29593242 PMC5871851

[3] Schumacher MA, Donnelly JM, Engevik AC, Xiao C, Yang L, Kenny S, Varro A, Hollande F, Samuelson LC, Zavros Y. Gastric sonic hedgehog acts as a macrophage chemoattractant during the immune response to *Helicobacter pylori*. Gastroenterology 142: 1150–1159.e6, 2012. doi:10.1053/j.gastro.2012.01.029. 22285806 PMC3335966

[4] Saha S, Aranda E, Hayakawa Y, Bhanja P, Atay S, Brodin NP, Li J, Asfaha S, Liu L, Tailor Y, Zhang J, Godwin AK, Tome WA, Wang TC, Guha C, Pollard JW. Macrophage-derived extracellular vesicle-packaged WNTs rescue intestinal stem cells and enhance survival after radiation injury. Nat Commun 7: 13096, 2016. doi:10.1038/ncomms13096. 27734833 PMC5065628

[5] Schumacher MA, Dennis IC, Liu CY, Robinson C, Shang J, Bernard JK, Washington MK, Polk DB, Frey MR. NRG4-ErbB4 signaling represses proinflammatory macrophage activity. Am J Physiol Gastrointest Liver Physiol 320: G990–G1001, 2021. doi:10.1152/AJPGI.00296.2020. 33826403 PMC8285586

[6] Zigmond E, Bernshtein B, Friedlander G, Walker CR, Yona S, Kim KW, Brenner O, Krauthgamer R, Varol C, Müller W, Jung S. Macrophage-restricted interleukin-10 receptor deficiency, but not IL-10 deficiency, causes severe spontaneous colitis. Immunity 40: 720–733, 2014. doi:10.1016/j.immuni.2014.03.012. 24792913

[7] Kamada N, Hisamatsu T, Okamoto S, Chinen H, Kobayashi T, Sato T, Sakuraba A, Kitazume MT, Sugita A, Koganei K, Akagawa KS, Hibi T. Unique CD14+ intestinal macrophages contribute to the pathogenesis of Crohn disease via IL-23/IFN-γ axis. J Clin Invest 118: 2269–2280, 2008. doi:10.1172/JCI34610. 18497880 PMC2391067

[8] Han X, Ding S, Jiang H, Liu G. Roles of macrophages in the development and treatment of gut inflammation. Front Cell Dev Biol 9: 625423, 2021. doi:10.3389/fcell.2021.625423. 33738283 PMC7960654

[9] Jayme TS, Leung G, Wang A, Workentine ML, Rajeev S, Shute A, Callejas BE, Mancini N, Beck PL, Panaccione R, McKay DM. Human interleukin-4-treated regulatory macrophages promote epithelial wound healing and reduce colitis in a mouse model. Sci Adv 6: eaba4376, 2020. doi:10.1126/sciadv.aba4376. 32548267 PMC7274799

[10] Callejas BE, Sousa JA, Flannigan KL, Wang A, Higgins E, Herik AI, Li S, Rajeev S, Rosentreter R, Panaccione R, McKay DM. Calcitonin gene-related peptide promotes epithelial reparative and anti-colitic functions of IL-4 educated human macrophages. Am J Physiol Gastrointest Liver Physiol 328: G1–G16, 2025. doi:10.1152/ajpgi.00159.2024. 39378308

[11] Ma W, Dumont Y, Vercauteren F, Quirion R. Lipopolysaccharide induces calcitonin gene-related peptide in the RAW264.7 macrophage cell line. Immunology 130: 399–409, 2010. doi:10.1111/j.1365-2567.2009.03239.x. 20141542 PMC2913219

[12] Lu YZ, Nayer B, Singh SK, Alshoubaki YK, Yuan E, Park AJ, Maruyama K, Akira S, Martino MM. CGRP sensory neurons promote tissue healing via neutrophils and macrophages. Nature 628: 604–611, 2024. doi:10.1038/s41586-024-07237-y. 38538784 PMC11023938

[13] Lewis JD, Parlett LE, Jonsson Funk ML, Brensinger C, Pate V, Wu Q, Dawwas GK, Weiss A, Constant BD, McCauley M, Haynes K, Yang JY, Schaubel DE, Hurtado-Lorenzo A, Kappelman MD. incidence, prevalence, and racial and ethnic distribution of inflammatory bowel disease in the United States. Gastroenterology 165: 1197–1205.e2, 2023. doi:10.1053/j.gastro.2023.07.003. 37481117 PMC10592313

[14] Le Berre C, Ricciuto A, Peyrin-Biroulet L, Turner D. Evolving short-and long-term goals of management of inflammatory bowel diseases: getting it right, making it last. Gastroenterology 162: 1424–1438, 2022. doi:10.1053/j.gastro.2021.09.076. 34995529

[15] Turner D, Ricciuto A, Lewis A, D’Amico F, Dhaliwal J, Griffiths AM, Bettenworth D, Sandborn WJ, Sands BE, Reinisch W, Schölmerich J, Bemelman W, Danese S, Mary JY, Rubin D, Colombel JF, Peyrin-Biroulet L, Dotan I, Abreu MT, Dignass A; International Organization for the Study of IBD. STRIDE-II: an update on the Selecting Therapeutic Targets in Inflammatory Bowel Disease (STRIDE) Initiative of the International Organization for the Study of IBD (IOIBD): determining therapeutic goals for treat-to-target strategies in IBD. Gastroenterology 160: 1570–1583, 2021. doi:10.1053/j.gastro.2020.12.031. 33359090

